# The Association of Telehealth Availability and Quality of Care Measures for Patients With Diabetes at Federally Qualified Health Centers: Retrospective Cohort Study

**DOI:** 10.2196/40827

**Published:** 2023-03-02

**Authors:** Megan B Cole, Nicholas Jones, Eun Kyung Lee, June-Ho Kim

**Affiliations:** 1 Boston University School of Public Health Boston, MA United States; 2 Brown University School of Public Health Providence, RI United States; 3 Ariadne Labs Harvard T H Chan School of Public Health Boston, MA United States; 4 Division of General Internal Medicine & Primary Care Brigham and Women’s Hospital Boston, MA United States

**Keywords:** telehealth, diabetes, quality of care, primary care, chronic disease, safety net, federally qualified health centers, COVID-19

## Introduction

Telehealth has the potential to improve access to and quality of care for low-income patients with chronic conditions such as diabetes [1-4]. This includes patients served by federally qualified health centers (FQHCs), which provide comprehensive primary and preventive care services to millions of low-income patients across the United States. In 2019, only 8% of FQHCs in Massachusetts were using telehealth to deliver primary care or chronic disease management services to patients [5]. By 2020, 100% of FQHCs in Massachusetts delivered at least some primary care or chronic disease care virtually via telehealth, with vast heterogeneity in the extent of adoption across FQHCs [5]. Our objective was to assess how FQHC-level use of telehealth was associated with access to and quality of care for low-income patients with diabetes at Community Care Cooperative (C3)—the largest FQHC-based Medicaid-accountable care organization (ACO) in the United States, which supported the adoption of telehealth delivery across FQHCs in Massachusetts during the COVID-19 pandemic [6].

## Methods

### Overview

Our study population included Medicaid-enrolled patients with a diagnosis of diabetes, aged 18-64 years, who were attributed to the C3 ACO, had at least 1 primary care visit to a C3 FQHC within the last 18 months, and otherwise met Healthcare Effectiveness Data and Information Set (HEDIS) denominator criteria for patients with diabetes (N=11,503 patient-months after exclusions). Our data source was the 2019-2021 Electronic Data Warehouse, which stores electronic health record (EHR) and claims data from C3; this included data from 11 FQHCs after excluding FQHCs with invalid EHR telehealth identifiers.

Study outcomes included three quality-of-care diabetes HEDIS measures (annual retinal exam, blood pressure control, blood glucose control) and a primary care engagement measure (number of FQHC visits). Our treatment group included FQHCs with high telehealth use during COVID-19 (≥50% of all visits mean 67.6%), and our comparison group included FQHCs with lower telehealth use during COVID-19 (<50% of all visits mean 36.7%). Telehealth included real-time video and audio-only visits delivered directly to remote patients.

Our unit of analysis was the patient-month. We used a difference-in-differences approach with linear probability models and negative binomial models to examine changes in patient-level outcomes for patients at high- versus low-telehealth FQHCs before (March 2019-February 2020) versus after (April 2020-March 2021) the rapid adoption of telehealth. All models adjusted for patient age, sex, and clinical risk score, applied month and FQHC fixed effects, used robust variance estimators, and applied inverse probability of treatment weights based on propensity scores to balance on 16 patient-level baseline covariates.

*P* values were 2-tailed, and statistical significance was set at *P*=.05. Analyses were performed using Stata 17.0 (StataCorp).

### Ethical Considerations

Boston University’s institutional review board deemed the study exempt because we used secondary deidentified data.

## Results

Of the 11,503 diabetic patient-months included in the study, the mean age was 48.1 years and 56.3% (n=6474) were female, with 27.3% (n=3142) being non-Hispanic White, 10.7% (n=1235) being non-Hispanic Black, and 53.4% (n=6139) being Hispanic based on self-reported race/ethnicity (Table 1). High telehealth use was associated with increases in 2 of 4 study outcomes, while 1 measure decreased (Table 2). Telehealth was associated with a relative 10.7 percentage point (95% CI 6.3-15.0; *P*<.001) increase in rates of annual retinal exams and a relative rate increase of 1.5 (95% CI 1.2-1.8; *P*<.001) in the number of visits among patients with diabetes, although overall visit rates fell in both groups during COVID-19. Telehealth was further associated with a 5.6 percentage point decrease (95% CI 9.7-1.5; *P*=.008) in the HEDIS blood pressure control measure and with no statistical change in the blood glucose control measure (*P*=.97).

**Table 1:** Baseline characteristics of patients with diabetes served at high vs low telehealth federally qualified health centers (FQHCs).

Characteristics^a^			Full sample (N=11,503 person-months, representing 1417 unique persons)		High telehealth^b^ (n=8196 person-months, representing 1022 unique persons)		Low telehealth^c^ (n=3307 person-months, representing 395 unique persons)		Standardized difference		
									Before propensity weighting		After propensity weighting
Age (years), mean (SD)			48.1 (11.4)		48.1 (11.5)		48.2 (11.1)		–0.0755		–0.0093
Sex (female), n (%)			6474 (56.3)		4823 (58.8)		1651 (49.9)		0.0889		0.0026
**Race/ethnicity^d^, n (%)**											
	White, non-Hispanic	3142 (27.3)		2337 (28.4)		805 (24.2)		0.1153		–0.0025	
	Black, non-Hispanic	1235 (10.7)		763 (9.4)		472 (14.4)		–0.4141		–0.0018	
	Hispanic	6139 (53.4)		4219 (51.6)		1920 (58.3)		0.1342		0.0010	
	Other or multirace	987 (8.6)		877 (10.6)		110 (3.2)		0.0936		0.0023	
**Primary language^e^, n (%)**											
	English	6447 (56.0)		4421 (54.0)		2026 (61.3)		0.0139		0.0114	
	Spanish	3954 (34.4)		2769 (33.8)		1186 (35.9)		0.0799		–0.0016	
	Other	1103 (9.6)		1006 (12.2)		97 (2.9)		–0.0851		–0.0074	
CDPS^f^ risk score, mean (SD)			3.65 (2.28)		3.56 (2.27)		3.90 (2.32)		0.1083		0.0054
**Clinical diagnoses, n (%)**											
	Hypertension	6557 (57.0)		4513 (55.1)		2044 (61.9)		–0.1115		0.0024	
	Hyperlipidemia	4039 (35.1)		3192 (38.8)		847 (25.4)		0.0644		–0.0106	
	Overweight or obese	6626 (57.6)		4789 (58.4)		1836 (55.5)		0.1284		–0.0118	
	Morbidly obese	2401 (20.9)		1855 (22.6)		546 (16.4)		0.2141		0.0013	
	Asthma	2388 (20.8)		1591 (19.4)		797 (24.2)		0.1134		0.0031	
	COPD^g^	1186 (10.3)		905 (11.0)		281 (8.5)		0.1254		0.0057	
	Tobacco use	3070 (26.7)		2192 (26.7)		878 (26.6)		0.0936		0.0153	
	Alcohol use disorder	1042 (9.1)		629 (7.7)		414 (12.6)		–0.0205		–0.0092	
	Major depression	4896 (42.6)		3853 (46.9)		1043 (31.3)		0.2817		–0.0048	
	Other depression	746 (6.5)		603 (7.3)		143 (4.3)		0.0843		–0.0032	
	Anxiety	3901 (33.9)		2976 (36.2)		925 (27.9)		0.2245		–0.0026	

^a^Baseline characteristics shown reflect characteristics in the unweighted sample. After applying inverse probability of treatment weights based on propensity scores, standardized differences between high vs low telehealth FQHCs for all 16 characteristics were less than 10%—a common threshold denoting negligible imbalance between groups.

^b^High telehealth includes patients at FQHCs where ≥50% of all visits to the FQHCs in the postperiod were delivered via telehealth (mean 67.6%), where telehealth includes real-time video and audio-only visits.

^c^Low telehealth includes patients at FQHCs where <50% of all visits to the FQHCs in the postperiod were delivered via telehealth (mean 36.7%), where telehealth includes real-time video and audio-only visits.

^d^Race/ethnicity of known (n=1983 person-months, 17.2% unknown race/ethnicity in full sample; n=1297 person-months, 15.9% unknown in high telehealth; and n=686 person-months, 20.8% unknown in low telehealth).

^e^Language of known (n=224, 2% unknown in full sample; n=196, 2.4% unknown in high telehealth; and n=28, 0.9% unknown in low telehealth).

^f^CDPS: Chronic Illness and Disability Payment System.

^g^COPD: chronic obstructive pulmonary disease.

**Table 2:** Association between federally qualified health center (FQHC)–level telehealth use and quality of care and visit volume among patients with diabetes.

Outcome^a^	High telehealth^b^		Low telehealth^c^		Adjusted coefficient^d^, DID^e^ (95% CI)		*P* value
	Pre	Post	Pre	Post			
Annual retinal exam, %	59.22	55.09	69.37	52.61	10.7 (6.29 to 15.0)	<.001	
Blood pressure control^f^, %	81.06	53.06	78.20	54.69	–5.57 (–9.67 to –1.47)	.008	
Blood glucose control^g^, %	67.33	51.54	63.26	47.66	–0.07 (–4.45 to 4.30)	.97	
Number of visits^h^	0.92	0.46	2.44	0.72	1.48 (1.24 to 1.77)	<.001	

^a^For all outcomes, 11503 person-months, representing 1417 unique patients. Sample sizes and analyses exclude the first 6 months of the postperiod due to the rolling, look-back nature of Healthcare Effectiveness Data and Information Set (HEDIS) measure calculation. For all outcomes, we tested the parallel trends assumption by interacting linear months with treatment status in the preperiod. No differential preperiod trends were detected (*P*>.05 for all).

^b^High telehealth includes patients at FQHCs where ≥50% of all visits to the FQHCs in the postperiod were delivered via telehealth (mean 67.6%), where telehealth includes real-time video and audio-only visits.

^c^Low telehealth includes patients at FQHCs where <50% of all visits to the FQHCs in the postperiod were delivered via telehealth (mean 36.7%), where telehealth includes real-time video and audio-only visits.

^d^Coefficients for HEDIS quality measures are from linear probability models, where coefficients are reported on an absolute percentage point scale; values >0 indicate that telehealth was associated with an increase in the measure. The coefficient for the number of visits is from a negative binomial model, where the coefficient is reported as a relative incidence rate ratio; values >1 indicate that telehealth was associated with an increase in the measure.

^e^DID: difference-in-differences.

^f^Blood pressure control indicates that a patient had a measurement documented within the last 12 months and a most recent result of <140/90 mmHg.

^g^Blood glucose control indicates that a patient had a measurement documented within the last 12 months and a most recent result ≤9.0%.

^h^The number of visits is measured as the mean number of FQHC visits per patient per month.

## Discussion

This study indicates that high FQHC-level telehealth use was associated with more sustained engagement in care for patients with diabetes at FQHCs in Massachusetts, with mixed effects on quality of care measures. Telehealth availability likely helped to partially mitigate the overall decrease in visit rates during COVID-19 by improving care access, while telehealth visits may have also provided the opportunity to encourage patients to return for in-person retinal screenings [7]. In contrast, decreases in measured blood pressure control may have resulted from the lack of standardized blood pressure documentation in EHRs during telehealth visits rather than poor control, although this was untestable in our data. While these findings fill an important evidence gap by focusing on Medicaid-enrolled FQHC patients, they are largely consistent with previous literature showing the potential for telehealth to improve access and some quality of care–sensitive outcomes in diabetic populations [8-10].

The results have important implications. First, telehealth has the potential to improve some measured quality, which suggests that incorporating telehealth into care delivery models and supporting telehealth sustainability through telehealth reimbursement may benefit patients with diabetes. Second, providers must simultaneously consider how to best adapt quality measures and underlying data reporting to ensure that quality-reliant payment models validly capture the quality of care under new delivery modalities, as measures such as blood pressure control may not be validly captured during telehealth encounters.

This research is not without limitations. Limitations include potential measurement error in the data, unmeasured confounding due to simultaneous implementation of telehealth and other service capacity changes during COVID-19, lack of subgroup analyses that explore heterogeneity in effects, and FQHC-level assignment of exposure. Nonetheless, based on this study, Medicaid reimbursement policies and care delivery models that support the use of telehealth as part of chronic disease care may be associated with better care engagement for low-income patients with diabetes.

## Abbreviations

ACO: accountable care organization
C3: Community Care Cooperative
EHR: electronic health record
FQHC: federally qualified health center
HEDIS: Healthcare Effectiveness Data and Information Set

## Data Availability

Data used for these analyses are governed by a data use agreement with Community Care Cooperative and all of its affiliated federally qualified health centers. Per the data use agreement, data are restricted and cannot be shared with outside parties. For further information regarding data availability, you may reach out to MBC at mbcole@bu.edu.
